# Molecular mechanisms underlying the neuroprotective effects of polyphenols: implications for cognitive function

**DOI:** 10.17179/excli2025-8779

**Published:** 2025-09-10

**Authors:** Justyna Godos, Giuseppe Carota, Giuseppe Caruso, Agnieszka Micek, Evelyn Frias-Toral, Francesca Giampieri, Julién Brito-Ballester, Carmen Lili Rodríguez Velasco, José L. Quiles, Maurizio Battino, Fabio Galvano, Giuseppe Grosso

**Affiliations:** 1 Department of Biomedical and Biotechnological Sciences (BIOMETEC), University of Catania, Catania 95123, Italy; 2 Departmental Faculty of Medicine, UniCamillus—Saint Camillus International University of Health Sciences, Rome 00131, Italy; 3 IRCCS San Camillo Hospital, Venice 30126, Italy; 4 Statistical Laboratory, Faculty of Health Sciences, Jagiellonian University Medical College, 31-501, Cracow, Poland; 5 Escuela de Medicina, Universidad Espíritu Santo, Samborondón, 0901952, Ecuador; 6 Division of Research, Texas State University, 601 University Dr, San Marcos, TX 78666, United States; 7 Department of Clinical Sciences, Università Politecnica delle Marche, 60131 Ancona, Italy; 8 Research Group on Food, Nutritional Biochemistry and Health, Universidad Europea del Atlántico, Isabel Torres 21, 39011 Santander, Spain; 9 Joint Laboratory on Food Science, Nutrition, and Intelligent Processing of Foods, Polytechnic University of Marche, Italy, Universidad Europea del Atlántico Spain and Jiangsu University, China at Polytechnic University of Marche, 60130 Ancona, Italy; 10 International Research Center for Food Nutrition and Safety, Jiangsu University, Zhenjiang 212013, China; 11 Universidad Internacional Iberoamericana Campeche 24560, México; 12 Faculty of Health Science, Universidad de La Romana. La Romana, República Dominicana; 13 Universidad Internacional Iberoamericana, Arecibo, Puerto Rico 00613, USA; 14 Faculty of Health Science, Universidade Internacional do Cuanza, Cuito, Bié, Angola; 15 Department of Physiology, Institute of Nutrition and Food Technology "José Mataix", Biomedical Research Centre, University of Granada, 18100 Granada, Spain

**Keywords:** polyphenols, neuroinflammation, blood-brain barrier, cerebral blood flow, neurotrophic factor

## Abstract

Polyphenols are naturally occurring compounds that can be found in plant-based foods, including fruits, vegetables, nuts, seeds, herbs, spices, and beverages, the use of which has been linked to enhanced brain health and cognitive function. These natural molecules are broadly classified into two main groups: flavonoids and non-flavonoid polyphenols, the latter including phenolic acids, stilbenes, and tannins. Flavonoids are primarily known for their potent antioxidant properties, which help neutralize harmful reactive oxygen species (ROS) in the brain, thereby reducing oxidative stress, a key contributor to neurodegenerative diseases. In addition to their antioxidant effects, flavonoids have been shown to modulate inflammation, enhance neuronal survival, and support neurogenesis, all of which are critical for maintaining cognitive function. Phenolic acids possess strong antioxidant properties and are believed to protect brain cells from oxidative damage. Neuroprotective effects of these molecules can also depend on their ability to modulate signaling pathways associated with inflammation and neuronal apoptosis. Among polyphenols, hydroxycinnamic acids such as caffeic acid have been shown to enhance blood-brain barrier permeability, which may increase the delivery of other protective compounds to the brain. Another compound of interest is represented by resveratrol, a stilbene extensively studied for its potential neuroprotective properties related to its ability to activate the sirtuin pathway, a molecular signaling pathway involved in cellular stress response and aging. Lignans, on the other hand, have shown promise in reducing neuroinflammation and oxidative stress, which could help slow the progression of neurodegenerative diseases and cognitive decline. Polyphenols belonging to different subclasses, such as flavonoids, phenolic acids, stilbenes, and lignans, exert neuroprotective effects by regulating microglial activation, suppressing pro-inflammatory cytokines, and mitigating oxidative stress. These compounds act through multiple signaling pathways, including NF-κB, MAPK, and Nrf2, and they may also influence genetic regulation of inflammation and immune responses at brain level. Despite their potential for brain health and cognitive function, polyphenols are often characterized by low bioavailability, something that deserves attention when considering their therapeutic potential. Future translational studies are needed to better understand the right dosage, the overall diet, the correct target population, as well as ideal formulations allowing to overcome bioavailability limitations.

See also the graphical abstract(Fig. 1).

## Introduction

In recent years, the relationship between diet and cognitive health has garnered significant attention due to the increasing global prevalence of neurodegenerative disorders, such as Alzheimer's disease (AD) and other forms of dementia (GBD 2016 Dementia Collaborators[100]). These conditions are characterized by progressive cognitive decline, memory loss, and impaired functional abilities, representing a major public health challenge worldwide (Lane et al., 2018[164]). As the aging population continues to rise, there is growing interest in understanding how dietary factors may contribute to brain health and potentially reduce the risk of cognitive decline (Zhang et al., 2021[296]). While genetic factors, environmental influences, and lifestyle choices have been widely studied, emerging evidence suggests that dietary components, particularly plant-derived phytochemicals, may play crucial roles in maintaining cognitive function, mitigating the onset of neurodegenerative diseases (Grabska-Kobylecka et al., 2023[112]).

The notion that diet can impact brain health is becoming increasingly clear based on growing evidence that certain dietary patterns, particularly those rich in plant-derived bioactive compounds, may hold promise in the prevention of cognitive decline (Cheng et al., 2022[57]). Phytochemicals are naturally occurring bioactive compounds found in plants that have been recognized for their health-promoting properties. These compounds, often categorized based on their chemical structure, possess a multimodal mechanism of action including antioxidant and anti-inflammatory activities allowing them to exert neuroprotection, influencing brain functions (Gentile et al., 2023[102]). They are commonly found in fruits, vegetables, nuts, seeds, and various plant-based foods (Rajaram et al., 2019[212]). There is convincing evidence that dietary patterns rich in plant-based foods, such as the Mediterranean diet, may exert positive effects on brain health and reduce the risk of cognitive decline and dementia (Fekete et al., 2025[90], Nucci et al., 2024[196]). Notably, while vitamins seem to play only a relative effect in the prevention of cognitive outcomes (Hu et al., 2025[129]), the main food groups involved in these putative effects are hypothesized to depend on their phytochemical components (Bavaro et al., 2024[24]).

Among the wide range of phytochemicals, polyphenols have gathered particular interest especially in relation to brain health (Castelli et al., 2018[48]). These compounds are abundant in fruits, vegetables, and beverages such as tea, coffee, and red wine (Roman et al., 2019[223]). Known for their potent antioxidant properties, polyphenols have been investigated for the potential modulatory effects on various molecular pathways involved in brain aging, inflammation, and neurodegenerative processes (Arias-Sanchez et al., 2023[17]). A growing body of observational studies investigating the neuroprotective effects of these compounds highlights their potential as therapeutic agents in promoting cognitive function and/or reducing the risk of cognitive decline (Godos et al., 2024[108]). Polyphenols have garnered attention for their ability to influence the central nervous system activities through multiple mechanisms (Meeusen et al., 2018[189]). These include modulating the activity of enzymes involved in neurotransmitter metabolism, promoting neurogenesis, modulating neuronal signaling, and reducing the accumulation of pathological protein aggregates that often represent hallmarks of neurodegenerative diseases, such as AD (Caruso et al., 2022[47]). In addition to their direct effects on brain cells, polyphenols may also exert systemic benefits by influencing systemic inflammation, oxidative stress, and vascular health, all of which are linked to cognitive function (Godos et al., 2024[108]).

Given the complexity of cognitive decline and the diverse roles that polyphenols compounds may play in brain health, it is critical to gain a comprehensive understanding of the mechanisms underlying their protective effects. In this context, this study aims to review the current scientific evidence on the relationship between polyphenols and cognitive health. The review will also discuss the potential mechanisms through which these bioactive compounds may contribute to the prevention of cognitive decline, with a focus on their molecular and cellular mechanisms, related but not limited to neuroinflammation, oxidative stress, and synaptic plasticity. Through this exploration, we aim to elucidate the potential for incorporating polyphenol-rich foods into dietary recommendations and therapeutic strategies for aging populations.

## Polyphenols: Definition and Classification

Polyphenols represent a large group of naturally occurring bioactive compounds characterized by the presence of multiple phenolic rings (Tsao, 2010[259]). These compounds are widely distributed in plants and are known for their antioxidant and anti-inflammatory properties (Pandey et al., 2009[205]). Polyphenols are divided into several subgroups based on their chemical structure, including flavonoids, phenolic acids, stilbenes, and lignans (Marin et al., 2015[186]). Each subclass has distinct chemical properties and exhibits different biological activities, although they share common mechanisms of action that may contribute to brain health (Tufarelli et al., 2017[260]). Flavonoids are one of the most studied groups of polyphenols and include a variety of subclasses divided according to their chemical structures in flavonols, flavones, isoflavones, anthocyanins, and flavan-3-ols (Manach et al., 2004[183]). Each compound has specific food sources (although a certain degree of overlap may occur) (Del Rio et al., 2013[75]). Flavonols, one of the most abundant subclasses of flavonoids, are characterized by a hydroxyl group at position 3 on the flavonoid ring (Wang et al., 2018[271]). These compounds, including quercetin, kaempferol, myricetin, and isorhamnetin, are widely distributed across a variety of fruits and vegetables, such as onions, especially red and yellow varieties, and apples (Aherne et al., 2002[5]). Flavones are characterized by a structure where the hydroxyl group is attached at position 4 of the flavonoid ring. This subclass includes compounds such as luteolin and apigenin, which are primarily found in herbs and vegetables (Harnly et al., 2006[120]). Luteolin is commonly present in celery, parsley, and artichokes, while apigenin is abundant in parsley, chamomile, and various citrus fruits (Dias et al., 2021[78]). Flavanols, which include catechins and epicatechins, represent another major subclass of flavonoids, best known for their presence in tea, cocoa, and certain fruits (Luo et al., 2022[181]). Green tea, in particular, is rich in epigallocatechin gallate (EGCG) (Khan et al., 2006[149]). Dark chocolate, made from cocoa beans, is another excellent source of flavanols, with a higher cocoa content offering greater levels of these beneficial compounds (Martin et al., 2021[187]). In addition to tea and chocolate, flavanols are also found in a variety of fruits including apples, grapes, and berries (Rudrapal et al., 2024[226]). Anthocyanins, another important subclass of flavonoids, are responsible for the red, purple, and blue pigmentation found in many fruits and vegetables (Mattioli et al., 2020[188]). These compounds include cyanidin, delphinidin, and malvidin, and are particularly prevalent in berries like blueberries, strawberries, blackberries, and raspberries. Other fruits such as cherries, plums, and grapes also provide significant amounts of anthocyanins (Lu et al., 2024[180]). Finally, isoflavones include genistein, daidzein, and glycitein, which are all known for their estrogenic properties, reason why they are also categorized as phytoestrogens (Zhang et al., 2022[298]). These compounds are typically contained in soy products (tofu, tempeh, and soy milk) and other legumes (Krizova et al., 2019[158]). Phenolic acids, including hydroxybenzoic and hydroxycinnamic acids, are another prominent class of polyphenols found in foods such as berries, coffee, whole grains, and some vegetables (Kumar et al., 2019[161]). Stilbenes, such as the well-known resveratrol abundant in red wine, and lignans, found in flaxseeds, sesame seeds, and whole grains, are less abundant than flavonoids and phenolic acids, but also play important roles in maintaining brain health (Rudrapal et al., 2024[226]). 

## Absorption, Metabolism, and Excretion of Polyphenols

The findings from preclinical and observational studies should be interpreted in light of current evidence that the bioavailability of polyphenols is generally low, with only a small fraction of ingested polyphenols absorbed into the bloodstream in their intact form (Di Lorenzo et al., 2021[77]). Several factors influence the absorption and distribution of polyphenols, including their chemical structure, solubility, interaction with food matrices, as well as the health and functional status of the digestive system (Rein et al., 2013[219]). The absorption of polyphenols begins in the mouth, where they may undergo preliminary enzymatic breakdown by salivary enzymes (Iqbal et al., 2023[134]). However, the primary processing of these compounds occurs in the stomach, where acidic conditions can aid in the dissolution of certain polyphenols (Iqbal et al., 2023[134]). For instance, phenolic acids found in foods like coffee and fruits are often more soluble under acidic conditions, allowing them to remain in a form that is more easily absorbed in the small intestine (Dama et al., 2024[71]). Nevertheless, only a small number of polyphenols is absorbed in the stomach due to the short contact with the gastric lining and the low permeability of the gastric epithelium (Crespy et al., 2002[68]). Thus, most of the absorption takes place in the small intestine (about 90 %) (Scalbert et al., 2002[229]). Here, polyphenols are absorbed via passive diffusion or active transport mechanisms, depending on their chemical characteristics (Brand et al., 2006[32]). Polyphenols are typically hydrophobic and may require the presence of carrier proteins or solubilizing agents, such as bile salts to facilitate absorption (Liu et al., 2023[176]). For example, flavonoids, which are typically glycosylated, must be hydrolyzed by enzymes such as β-glucosidases before being absorbed (Day et al., 2000[73]), entering the bloodstream, and being transported to the liver via the portal vein. Upon reaching the liver, absorbed polyphenols undergo phase I and phase II metabolism (Cardona et al., 2013[38], Stevens et al., 2016[248]). Phase I reactions typically involve oxidation, reduction, or hydrolysis of the polyphenolic compounds, leading to the formation of more hydrophilic metabolites (Kroon et al., 2004[159]). Enzymes such as cytochrome P450 (CYP450) play a key role in these transformations, leading to the production of hydroxylated and methylated metabolites (Hodek et al., 2002[125], Otake et al., 2002[202]). These phase I metabolites may exhibit altered activity compared to the parent compounds (Chen et al., 2014[54], Lampe et al., 2007[163]). Following phase I metabolism, polyphenols undergo phase II conjugation reactions, which involve the addition of hydrophilic groups, and include processes such as glucuronidation, sulfation, acetylation, and glutathione conjugation (Crozier et al., 2010[69]). These conjugated compounds are typically more water-soluble, which facilitates their elimination from the body (Chen et al., 2005[50], Kroon et al., 2004[159], Kuhnle et al., 2000[160]). For example, after the consumption of quercetin or epicatechins the liver typically conjugates these compounds with glucuronic acid or sulfate, forming quercetin glucuronide or epicatechin sulfate (Actis-Goretta et al., 2012[2], Ottaviani et al., 2012[203]). These metabolites are then released into the bloodstream and distributed to various tissues and organs (Carrillo-Martinez et al., 2024[41]).

Microbial metabolism of polyphenols results in the production of various metabolites, which can have beneficial effects on host health (Mithul Aravind et al., 2021[193]). For instance, the breakdown of flavonoids like anthocyanins by gut bacteria produces bioactive phenolic acids such as protocatechuic and syringic acids, which can be absorbed and exert systemic effects (Burdulis et al., 2009[33], Keppler et al., 2005[148], Miladinovic et al., 2014[192]). Similarly, the microbial metabolism of lignans contained in foods like flaxseeds can lead to the formation of enterolignans, which have been linked to reduced risk of certain chronic diseases, including metabolic syndromes and cardiovascular diseases (Frankenfeld, 2014[95]). The gut microbiota is also able to influence the bioavailability of polyphenols through the modification of their chemical structure, the degradation of glycosides, or the transformation of complex compounds into simpler forms that could be easily absorbed (Kumar Singh et al., 2019[162], Manach et al., 2004[183]). Additionally, the diversity and composition of the gut microbiota can significantly impact the individual ability to metabolize polyphenols, which may contribute to variations in individual responses to dietary polyphenols (Favari et al., 2024[89]).

After absorption and hepatic metabolism, polyphenolic metabolites circulate in the bloodstream, where they are distributed to various organs, including the brain (Vauzour, 2012[264]). However, the extent to which these metabolites reach target tissues and exert their effects depends on their physicochemical properties and the efficiency of transport mechanisms (Suominen et al., 2015[250], Youdim et al., 2003[286], 2004[287]). Polyphenols, especially those with smaller molecular size or increased hydrophilicity through conjugation, are able to cross the blood-brain barrier (BBB), which selectively allows the passage of substances into the central nervous system (Youdim et al., 2003[286], 2004[287]). For instance, resveratrol has been shown to cross the BBB and exert neuroprotective effects in animal models (Huang et al., 2011[130], Liu et al., 2012[172], Wang et al., 2002[269]). Other polyphenolic compounds, such as quercetin and epicatechin, are also able to reach and accumulate in the brain and have been associated with improved cognitive function and reduced neuroinflammation (Chiang et al., 2023[60], Wrobel-Biedrawa et al., 2022[275], Zhao et al., 2022[301]).

As previously mentioned, the liver plays a central role in the metabolism of polyphenols, as it is the primary site of phase I and phase II metabolism (Anderle et al., 2004[13], Rechner et al., 2002[215]). Once the liver has processed these compounds, they may be excreted in bile or enter the bloodstream for distribution to other organs. In contrast, the kidneys are the primary organs responsible for the excretion of polyphenolic metabolites via urine (Clarke et al., 2022[62], Crespy et al., 2003[67]). For instance, after the ingestion of polyphenols like catechins or ellagic acid, their metabolites, such as methylated or glucuronidated derivatives, are typically excreted in the urine (Clifford et al., 2013[63]). The kidneys, therefore, play a crucial role in eliminating excess polyphenols and their metabolites from the body, although small amounts may also be excreted via bile or feces (Crozier et al., 2010[69]). The efficiency of excretion depends on the chemical structure of the polyphenols and the modifications made by the liver and gut microbiota. Generally, water-soluble conjugates are more readily excreted in the urine, while lipophilic polyphenols may be excreted in bile or undergo enterohepatic recycling. Some polyphenols are extensively metabolized and excreted rapidly, while others may persist in the body for longer periods before being eliminated. For example, catechins from green tea are rapidly absorbed, metabolized, and excreted, whereas stilbenes such as resveratrol are known to have a longer half-life in the body and may accumulate in tissues before being eliminated (Crozier et al., 2010[69]).

## Potential Mechanisms of Action of Dietary Polyphenolinfluencing Cognitive Health

### Antioxidant activity of polyphenols

**Figure 2:**
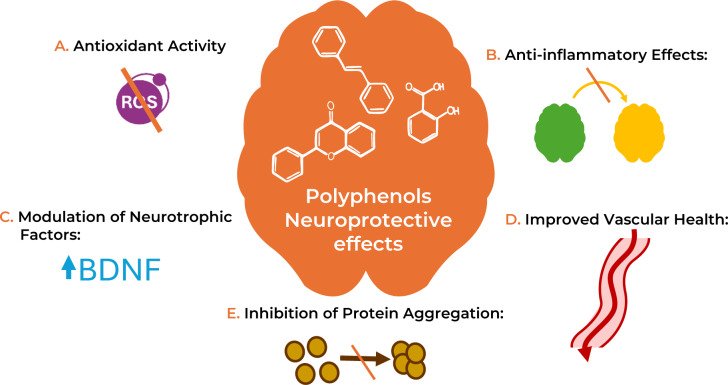
Mechanisms of action of polyphenols on cognitive health. The neuroprotective effects of polyphenols occur through multiple mechanisms, including: (A) Antioxidant activity: polyphenols neutralize free radicals and reactive species, including ROS, counteracting oxidative stress; (B) Anti-inflammatory effects: polyphenols modulate inflammatory signaling pathways, including the inhibition of pro-inflammatory cytokines and enzymes such as cyclooxygenase-2 (COX-2) and inducible nitric oxide synthase (iNOS); (C) Modulation of neurotrophic factors: polyphenols have been shown to enhance the expression of brain-derived neurotrophic factor (BDNF), a protein that promotes neurogenesis and synaptic plasticity. BDNF is crucial for the growth, maintenance, and survival of neurons, particularly in areas involved in memory and learning, such as the hippocampus; (D) Improved vascular health: polyphenols can improve endothelial function and promote vasodilation, which enhances blood flow to the brain. Better cerebrovascular health may improve the delivery of oxygen and nutrients to brain cells, contributing to cognitive function and reducing the risk of ischemic-related cognitive decline; (E) Inhibition of protein aggregation: some polyphenols have been shown to inhibit the aberrant aggregation of proteins, thereby potentially inhibiting the progression of disease such as AD.

Oxidative stress is a condition where the production of pro-oxidant mediators, such as reactive oxygen species (ROS), exceeds the body's antioxidant defenses, leading to cellular damage (Caruso et al., 2017[44], Sies, 2015[238]). In the brain, oxidative stress significantly contributes to neuroinflammation, neurodegeneration, and cognitive decline phenomena, all features of neurodegenerative diseases like AD (Teleanu et al., 2022[255]). One mechanism through which polyphenols may exert neuroprotective effects is by acting as antioxidants and preventing oxidative stress in the brain (Gilgun-Sherki et al., 2001[104], Lee et al., 2020[167], Pisoschi et al., 2015[208]) (Figure 2(Fig. 2)).

Antioxidants work by neutralizing ROS, which include free radicals such as superoxide anions (O_2_^•^^−^), hydroxyl radicals (• OH), and non-radical species like hydrogen peroxide (H_2_O_2_). ROS are generated as by-products of normal cellular metabolism, but their levels can increase under conditions of cellular stress, leading to neuroinflammation or neurodegeneration (Yang et al., 2020[283]). Polyphenols act as antioxidants primarily through their ability to donate electrons or hydrogen atoms to ROS, thereby stabilizing these reactive species and preventing them from causing cellular damage (Dias et al., 2021[78]). Many polyphenols, particularly flavonoids (e.g., quercetin, catechins, and anthocyanins), possess hydroxyl groups (-OH) on their chemical structure, which are capable of scavenging ROS by donating hydrogen atoms (Dias et al., 2021[78]). This neutralization of free radicals is one of the primary antioxidant mechanisms through which polyphenols exert protective effects. For instance, quercetin is known to directly scavenge O_2_^•^^−^ and •OH, reducing oxidative stress in brain cells (Cheng et al., 2024[56], Ho et al., 2022[124]). In addition to directly scavenging ROS, polyphenols can also modulate the expression of antioxidant enzymes (Al-Khayri et al., 2022[10]). One key target is nuclear factor erythroid 2-related factor 2 (Nrf2), a transcription factor that regulates the expression of genes involved in antioxidant response (Alavi et al., 2021[7], Kim et al., 2020[153]). Upon activation, Nrf2 binds to antioxidant response elements (AREs) in the promoter regions of genes encoding for enzymes like superoxide dismutase (SOD), catalase, and glutathione peroxidase (GPx). These enzymes are crucial for neutralizing ROS, protecting cells from oxidative damage (Alavi et al., 2021[7]). Polyphenols such as resveratrol, curcumin, and epicatechins have been shown to activate Nrf2, enhancing the brain's antioxidant defense system and reducing oxidative stress (Duan et al., 2022[82], Han et al., 2014[118], Zamanian et al., 2023[290], Zhang et al., 2020[300]). Polyphenols may also reduce oxidative stress by inhibiting the activity of enzymes that generate ROS (Darwish et al., 2023[72]). For instance, polyphenols like resveratrol have been shown to inhibit the activity of NADPH oxidase (NOX) enzymes, which are involved in the generation of O_2_^•^^−^ (Bagul et al., 2015[21], Cheng et al., 2014[58]). Inhibiting the activity of these enzymes, polyphenols prevent the excessive production of ROS and reduces oxidative damage in brain cells.

Despite the plethora of studies supporting such direct or indirect antioxidant effects of dietary polyphenols on brain cells, current evidence suggests that only few compounds reach the central nervous system avoiding transformation (Grabska-Kobylecka et al., 2023[112]). In fact, the BBB is a selective barrier that tightly regulates the passage of substances from the bloodstream into the brain (Campos-Bedolla et al., 2014[35]). While the BBB protects from harmful substances, it also limits the ability of many therapeutic compounds, including polyphenols, to reach the brain (Genchi et al., 2024[101]). This raises important questions about how polyphenols, which have been shown to exert antioxidant effects in peripheral tissues, can exert similar activities in the brain. In fact, aside from the aforementioned general low bioavailability of polyphenols, some compounds are also hydrophilic (water-soluble) (Aditya et al., 2017[4]), characteristic that reduces the ability to cross the lipid-rich BBB. The ability of certain polyphenols for which the strongest evidence of the effects on brain is available, such as anthocyanins, is rather limited and depends on several factors, including their chemical form, metabolism, and the presence of transport mechanisms (Godos et al., 2025[106]).

Concerning anthocyanins specifically, these compounds are less likely to cross the BBB in their native form due to their size and polarity: however, their metabolites (such as, phenolic acids) that are smaller and more lipophilic, can cross the BBB more easily (Godos et al., 2025[107]). Also, natively smaller polyphenols, such as resveratrol, quercetin, and epicatechins, can cross the BBB to some extent (Azargoonjahromi et al., 2024[20], Deepika et al., 2022[74], Faria et al., 2011[87]), most probably through passive diffusion.

Resveratrol is one of the most widely studied polyphenols for its therapeutic potential in the central nervous system (Azargoonjahromi et al., 2024[20]). Different studies have shown that resveratrol can reach the brain following oral administration, despite a low bioavailability due to rapid metabolism in the liver and intestines (Andrade et al., 2018[16], Francioso et al., 2014[94]). Once in the brain, resveratrol exerts antioxidant effects by activating Nrf2 and inhibiting inflammatory pathways (Farkhondeh et al., 2020[88], Yang et al., 2023[282]). Other mechanisms of direct counteraction of oxidative stress include involvement of catalase, SOD, GPx, glutathione reductase, as well as glutathione (GSH) content, while the levels of pro-inflammatory factors, such as IL-1β and IL-6 are downregulated (Zhang et al., 2020[300]). Besides, preclinical studies also showed that resveratrol may protect the basement membrane tight junction proteins to improve BBB integrity attenuating the expression of Intercellular Adhesion Molecule 1 (ICAM-1) and Vascular Cell Adhesion Molecule 1 (VCAM-1) and reduce the expression of pro-inflammatory genes such as iNOS and IL-1β, as well as increasing the level of anti-inflammatory factors, including arginase 1 and cytokine IL-10 (Wang et al., 2016[266]).

Flavonoids, such as quercetin and epicatechin, have been shown to cross the BBB in animal studies, although the quantities that reach the brain are often small (Assuncao et al., 2015[18]). The passage of flavonoids across the BBB is thought to be facilitated by their relatively small molecular size and their ability to interact with transporters of the endothelial cells that form the BBB (Carecho et al., 2021[39]). Such molecules have been shown to regulate the expression of Nrf2 and related pathways (Smith et al., 2016[242]). Activation of Nrf2 by flavonoids induces the expression of antioxidant enzymes, such as SOD, catalase, and heme oxygenase-1 (HO-1), which are devoted to control and/or mitigate oxidative stress (Owjfard et al., 2024[204], Smith et al., 2016[242]). Studies in rodents have demonstrated that quercetin can reach brain regions such as the hippocampus and cortex, where it can exert antioxidant and anti-inflammatory effects (AbdElrazek et al., 2023[1], Du et al., 2022[81], Jiao et al., 2023[140], Zargar et al., 2021[292]). 

### Polyphenols and neuroinflammation

**Figure 3:**
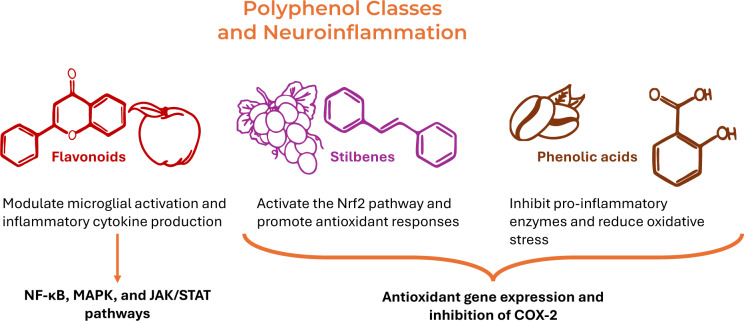
Differential Effects of Polyphenol Classes on Neuroinflammation. While all polyphenols share the ability to modulate neuroinflammation, the specific mechanisms of action can differ between the different classes. Flavonoids tend to be more effective at modulating microglial activation and inflammatory cytokine production, while phenolic acids often act through inhibition of pro-inflammatory enzymes and oxidative stress reduction. Stilbenes, like resveratrol, are particularly effective at activating the Nrf2 pathway and promoting antioxidant responses. Flavonoids like quercetin and catechins are potent modulators of NF-κB, MAPK, and JAK/STAT pathways, while resveratrol and phenolic acids focus more on antioxidant gene expression and inhibition of COX-2.

Neuroinflammation, the inflammation of the brain and spinal cord, is a key pathological feature of different neurodegenerative diseases such as AD (Teleanu et al., 2022[255]). It is primarily driven by the activation of microglia and astrocytes that release pro-inflammatory cytokines leading to oxidative stress and the disruption of neuronal homeostasis (Giri et al., 2024[105]). Microglia are the resident immune cells of the brain, and their activation plays a central role in neuroinflammation (Cardaci et al., 2025[37], Wang et al., 2023[265]). Under normal conditions, microglia are involved in immune surveillance and maintenance of homeostasis reacting to external stimuli by releasing pro-oxidant mediators and pro-inflammatory cytokines (such as TNF-α, IL-1β, and IL-6), also activating processed that, when resolved, can damage neurons (Isik et al., 2023[135], Wang et al., 2023[265]). While neuroinflammation plays a protective role during acute injury or infection, chronic neuroinflammation is detrimental and can contribute to the progression of neurological diseases (Rajesh et al., 2022[213]). Increasing evidence suggests that dietary polyphenols exert significant anti-inflammatory effects on the brain, making them promising candidates for the prevention and/or treatment of neurodegenerative conditions (Borda et al., 2025[30], Grabska-Kobylecka et al., 2023[112]). Their mechanisms of action in neuroinflammation are multifaceted, including the modulation of inflammatory pathways, suppression of oxidative stress, and regulation of gene expression (Arias-Sanchez et al., 2023[17]) (Figure 3(Fig. 3)).

Several studies have shown that flavonoids can inhibit microglial over-activation (Chen et al., 2022[52]). For instance, quercetin, a widely studied flavonoid, has been shown to suppress the production of pro-inflammatory cytokines in activated microglia by inhibiting the nuclear factor kappa B (NF-κB) signaling pathway (Li et al., 2023[168]), a key transcription factor that regulates the expression of genes involved in inflammation. Inhibition of NF-κB signaling by quercetin results in decreased expression of pro-inflammatory mediators including TNF-α, IL-1β, and cyclooxygenase-2 (COX-2), thus attenuating neuroinflammation (Adeoluwa et al., 2023[3], Bahar et al., 2017[22]). Similarly, other flavonoids, such as epicatechin (found in cocoa) and catechins (found in green tea), exhibit anti-inflammatory properties by modulating microglial activity (Chen et al., 2022[49], Regan et al., 2024[217]). Flavonoids are also able to regulate additional key signaling pathways involved in neuroinflammation. The mitogen-activated protein kinase (MAPK) pathway is mediating inflammation in microglia: the MAPK family includes extracellular signal-regulated kinase (ERK), c-Jun N-terminal kinase (JNK), and p38 MAPK, all of which contribute to the production of pro-inflammatory cytokines (Ten Bosch et al., 2021[256]). Studies have shown that flavonoids like quercetin can inhibit p38 MAPK and JNK, thus reducing the inflammatory response in microglial cells (Huang et al., 2023[131]), in accordance with the ability of flavonoids to modulate the expression of genes involved in the regulation of immune responses (Kim et al., 2020[153]). Additional studies have shown that apigenin and luteolin can downregulate the expression of pro-inflammatory cytokines (e.g., TNF-α, IL-1β) in activated microglia and astrocytes, partly through the modulation of transcription factors such as NF-κB and activator protein 1 (AP-1) (Chen et al., 2020[51], Kempuraj et al., 2021[146], Xie et al., 2023[278]).

Phenolic acids, such as ferulic, caffeic, and ellagic acids, are common polyphenols found in coffee, nuts, grains, and certain fruits and vegetables (Caruso et al., 2022[46]). As observed for flavonoids, phenolic acids have been shown to regulate neuroinflammation, albeit through slightly different mechanisms (Caruso et al., 2022[46]). These compounds can inhibit the activity of pro-inflammatory enzymes, such as COX and lipoxygenase (LOX), which are involved in the production of pro-oxidant and pro-inflammatory mediators like prostaglandins and leukotrienes (Szwajgier et al., 2017[251]). In particular, caffeic acid has been shown to inhibit COX-2 expression and reduce the production of prostaglandin E2 (PGE2), a potent pro-inflammatory mediator (Kang et al., 2009[145]). By suppressing the activity of these enzymes, phenolic acids can reduce neuroinflammation counteracting neurodegenerative processes. Phenolic acids can also inhibit the NF-κB signaling pathway, thereby reducing the production of pro-inflammatory mediators (Caruso et al., 2022[46]). For instance, ferulic acid has been shown to reduce the activation of NF-κB in microglia, leading to decreased expression of inflammatory cytokines such as IL-1β and TNF-α (Rehman et al., 2019[218]). This reduction in NF-κB activation is considered one of the key mechanisms by which phenolic acids exert their neuroprotective effects.

As previously mentioned, resveratrol is one of the most widely studied polyphenols for its neuroprotective and anti-inflammatory effects (Galiniak et al., 2019[98], Islam et al., 2022[136]). Resveratrol exerts its anti-inflammatory effects through a variety of mechanisms, including modulation of microglial activation, inhibition of NF-κB signaling, and activation of sirtuin 1 (SIRT1), a protein that regulates inflammatory responses and cellular stress (Meng et al., 2021[191], Wu et al., 2020[277]). Resveratrol has been shown to reduce the release of pro-inflammatory cytokines and nitric oxide (NO) from activated microglia and astrocytes (Fan et al., 2021[85], Liu et al., 2022[174], Omraninava et al., 2021[198]). It also inhibits the formation of the inflammasome, a multiprotein complex that plays a critical role in the activation of caspase-1 and the processing of pro-inflammatory cytokines such as IL-1β (Schlotterose et al., 2023[231]). By inhibiting these inflammatory pathways, resveratrol reduces neuroinflammation and protects neurons from degeneration. The Janus kinase (JAK)/signal transducer and activator of transcription (STAT) pathway is another key regulator of neuroinflammation: JAK/STAT activation promotes the transcription of genes associated with inflammation, such as IL-6 and IL-12 (Hu et al., 2023[128], Xin et al., 2020[279]). Polyphenols like resveratrol have been shown to inhibit the JAK/STAT pathway in activated glial cells, leading to reduced expression of pro-inflammatory cytokines and protection of neuronal function (Ji et al., 2024[138], Zhang et al., 2024[293]). 

Lignans, such as those found in flaxseeds, sesame seeds, and whole grains, have also been shown to possess anti-inflammatory properties (Singh et al., 2023[240]). Lignans such as enterolactone and enterodiol modulate the expression of pro-inflammatory cytokines and enzymes and exhibit antioxidant activity (Johnson et al., 2019[142]). Like flavonoids and phenolic acids, lignans inhibit NF-κB and MAPK signaling pathways, helping to alleviate, or in the best scenario prevent, chronic neuroinflammation (Wang et al., 2024[270], Yang et al., 2024[284]).

### Interaction between dietary polyphenols and the gut microbiota

The human gastrointestinal (GI) tract contains a diverse and complex community of microorganisms, collectively referred to as the gut microbiota (Golshany et al., 2025[111]). These microorganisms, which include bacteria, fungi, viruses, and archaea, play a pivotal role in human health, particularly in the digestion of food, metabolism of nutrients, and modulation of immune and inflammatory responses (Kamada et al., 2013[143], Rowland et al., 2018[225]). Also, there is growing evidence suggesting that gut microbiota may exert direct regulating effects toward brain via a gut-brain axis (Ayyanar et al., 2025[19]). Recent research has highlighted the importance of the gut microbiota in influencing the absorption, metabolism, and bioactivity of dietary polyphenols (Favari et al., 2024[89]). These compounds, being largely non-digestible by human enzymes, pass into the colon, where they interact with the gut microbiota that, in turn, transforms these polyphenols into a wide range of metabolites that can be absorbed into the bloodstream and exert systemic effects (Narduzzi et al., 2022[195]). Understanding these interactions and the influence of polyphenols on gut microbial composition and diversity is then crucial for comprehending how dietary patterns rich in polyphenols may impact human health (Favari et al., 2024[89]).

The interaction between dietary polyphenols and gut microbiota can vary significantly depending on their chemical structure, solubility, and metabolism (Zhang et al., 2022[299]). Generally, polyphenols that reach the colon unabsorbed by the small intestine become part of the microbial fermentation process (Kilua et al., 2022[150]). Microorganisms in the colon, particularly gut bacteria, possess a range of enzymes capable of breaking down the complex polyphenolic structures into smaller, bioactive metabolites (Alqudah et al., 2024[11]). These metabolites may have a profound effect on both gut microbiota composition and host health (Rana et al., 2022[214]). Some mechanisms have been hypothesized to explain how dietary polyphenols might influence gut microbiota. Certain polyphenols can directly impact the growth and activity of specific microbial populations (Sorrenti et al., 2020[246]). For instance, some polyphenols exhibit antimicrobial properties (Nyiew et al., 2022[197]), which can suppress the overgrowth of pathogenic bacteria or reduce microbial dysbiosis (an imbalance in the gut microbiota). Conversely, some polyphenols serve as substrates that selectively promote the growth of beneficial microbes (Rodriguez-Daza et al., 2021[221]). In fact, polyphenols undergo enzymatic breakdown by gut microbiota to produce metabolites, such as phenolic acids, aromatic compounds, and short-chain fatty acids (SCFAs) (Zhang et al., 2024[295]). These metabolites may have prebiotic effects, promoting the growth of beneficial gut bacteria and inhibiting the growth of pathogenic bacteria. Moreover, they have also been shown to improve gut health, reduce inflammation, and support the integrity of the intestinal barrier (Cheng et al., 2023[55]). In fact, the metabolites produced by the gut microbiota upon polyphenols' fermentation can influence immune responses in the gut. For example, some microbial metabolites have shown to modulate the expression of pro-inflammatory cytokines (Kiriyama et al., 2024[155]), thus playing a role in systemic and gut-specific inflammation. Finally, polyphenols may interact with microbial enzymes or signal transduction pathways that regulate the expression of genes involved in fermentation and metabolism, thus influencing microbial activity and composition.

While the exact mechanisms are still under investigation, numerous studies have provided evidence supporting the ability of polyphenols to significantly influence the composition and diversity of gut microbiota (Marchesi et al., 2016[185]). The impact of polyphenols on gut microbiota appears to be compound-specific, with differences observed between polyphenol subclasses such as flavonoids, phenolic acids, stilbenes, and lignans. Flavonoids, such as quercetin, catechins, and anthocyanins have been shown to modulate the abundance of various bacterial groups (Shabbir et al., 2021[236]). For example, studies have reported an increase in beneficial bacterial families such as *Lactobacillus* and *Bifidobacterium*, which are associated with gut health and the production of SCFAs (Lin et al., 2019[171]), improving gut barrier function and reducing intestinal inflammation. Conversely, flavonoids may reduce the population of pathogenic bacteria, such as *Enterococcus* and *Clostridium* species, which are associated with gut dysbiosis and inflammation. A number of studies have also shown that flavonoid-rich diets (e.g., consumption of fruits and vegetables or tea) can increase gut microbiota diversity (Sakkas et al., 2020[227]). Increased microbial diversity is generally considered a marker of a healthy gut microbiome, as it is associated with a more resilient and balanced microbial community. For instance, dietary intake of flavonoids from foods like apples and onions has been linked to an increase in the overall microbial richness of the gut. Many flavonoids have been shown to possess prebiotic-like properties (Taherkhani et al., 2024[252], Wang et al., 2022[268]). The gut microbiota metabolizes these compounds into smaller phenolic acids that act as selective substrates for beneficial microbes. 

Phenolic acids represent another major subclass of polyphenols. The microbial metabolism of phenolic acids, particularly hydroxycinnamic and hydroxybenzoic acids, plays an essential role in modulating gut microbiota composition (Loo et al., 2020[178]). Hydroxycinnamic acids, such as caffeic acid and ferulic acid, are fermented by gut microbiota into bioactive metabolites that have been shown to promote the growth of beneficial bacterial groups, including *Lactobacillus*, *Bifidobacterium*, and *Akkermansia muciniphila*. These bacteria are involved in the fermentation of dietary fiber and the production of SCFAs like butyrate, which possesses anti-inflammatory properties and supports intestinal health (Liu et al., 2018[173]). Moreover, phenolic acids, particularly those derived from berries and grains, can suppress the growth of harmful microorganisms, such as *Bacteroides *(Yu et al., 2024[289]). These microbes have been linked to conditions such as gut dysbiosis and chronic inflammatory diseases (Serino, 2018[234]), and their suppression by polyphenol metabolites may then contribute to improved gut health. These smaller bioactive compounds, such as valeric, phenylpropionic, and hydroxyphenylacetic acids, have been shown to exert anti-inflammatory, antioxidant, and antimicrobial effects, contributing to improved gut health and immune modulation.

Studies on animal models observed that resveratrol was able to modulate the gut microbiota by increasing the abundance of beneficial microbes including *Lactobacillus* and *Bifidobacterium* (Yao et al., 2022[285]). In addition to the above, results have demonstrated that resveratrol may reduce the abundance of pathobionts such as *Firmicutes* and *Proteobacteria*, the activity of which has been associated with inflammation and metabolic disorders (Yao et al., 2022[285]). Lignans, found in flaxseeds and sesame seeds, are converted by gut bacteria into enterolignans like enterodiol and enterolactone (Kleigrewe et al., 2022[156]). These metabolites have been shown to exert antioxidant and anti-inflammatory effects, influencing the gut microbiota by promoting the growth of beneficial bacteria while inhibiting harmful ones (Parikh et al., 2019[206]). Enterolignans also contribute to the modulation of estrogen metabolism and may have protective effects against hormone-related cancers.

### Modulation of neurotrophic factors by polyphenols 

**Figure 4:**
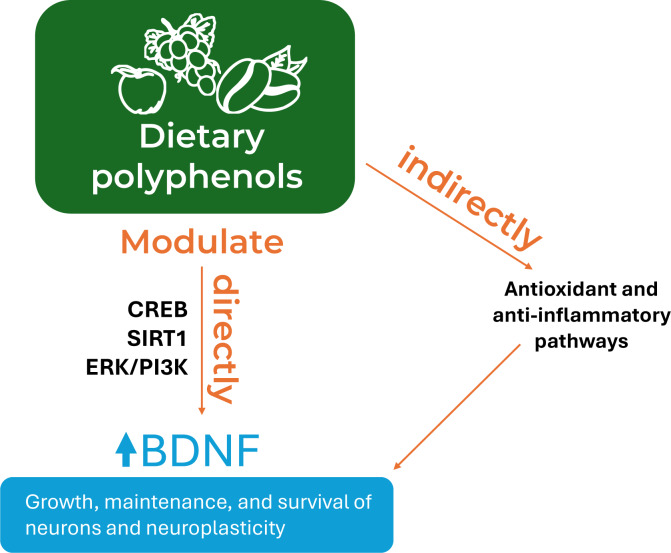
Modulation of neurotrophic factors by dietary polyphenols. Dietary polyphenols modulate the expression of BDNF in the brain, particularly in regions involved in learning and memory, such as the hippocampus. This regulation of BDNF contributes to the growth, maintenance, and survival of neurons and promotes neuroplasticity, which is crucial for cognitive function. The specific mechanisms through which polyphenols exert these effects depend on the class of polyphenol, with flavonoids, phenolic acids, and stilbenes activating different signaling pathways such as CREB, SIRT1, and ERK/PI3K. Furthermore, polyphenols' antioxidant and anti-inflammatory effects may also indirectly support BDNF expression, particularly in the context of neurodegenerative diseases.

Neurotrophic factors are essential molecules that promote the growth, maintenance, survival, and differentiation of neurons, also playing a crucial role in regulating neuroplasticity, the brain's ability to reorganize itself by forming new neural connections (Liu, 2018[175]). One of the most well-known and studied neurotrophic factors is the brain-derived neurotrophic factor (BDNF), which plays a key role in neuronal development, synaptic plasticity, and cognitive functions including learning and memory (Colucci-D'Amato et al., 2020[64]). BDNF exerts its effects through the activation of two primary receptors: TrkB (tropomyosin receptor kinase B) and p75NTR (low-affinity neurotrophin receptor) (Leal et al., 2014[166], Lu et al., 2014[179]). Activation of TrkB by BDNF promotes intracellular signaling cascades that lead to neuronal survival, synaptic growth, and enhanced plasticity. On the other hand, p75NTR activation is involved in apoptosis and cell death under certain conditions, and its balance with TrkB signaling is important for neuronal homeostasis (Gao et al., 2017[99]). Dysregulation of BDNF expression is implicated in various neurodegenerative diseases, mood disorders, and cognitive impairments, making it an important target for therapeutic strategies. Recent research has shown that dietary polyphenols can modulate the expression of BDNF, thereby enhancing neuronal survival and function (Carrillo et al., 2019[40]) (Figure 4(Fig. 4)).

The modulation of neurotrophic factors represents one of the mechanisms through which polyphenols exert neuroprotective effects and may contribute to the prevention and/or inhibition of the development of neurodegenerative diseases such as AD and PD. One of the most studied pathways through which flavonoids regulate BDNF expression is the cAMP response element-binding protein (CREB) pathway (Sharma et al., 2019[237]). CREB is a transcription factor that plays a crucial role in the expression of genes involved in memory and learning, including BDNF. Flavonoids like quercetin (Grewal et al., 2021[113]) and epicatechin (Rothenberg et al., 2019[224]) have been shown to activate CREB, leading to increased transcription of BDNF and other neuroprotective genes (Mehranfard et al., 2023[190], Younis et al., 2024[288], Zhang et al., 2019[294]). In rodent models, 3,5,6,7,8,3',4'-Heptamethoxyflavone (HMF), a citrus polymethoxyflavone, has been shown to increase BDNF levels in the hippocampus, and this increase is linked to improvements in learning and memory. HMF activates the CREB pathway, which in turn stimulates the transcription of BDNF. This is particularly important for synaptic plasticity and cognitive function. Another mechanism involves regulation of histone deacetylases (HDACs), which are enzymes that repress gene expression by removing acetyl groups from histones, making DNA more tightly packed and less accessible to transcription factors (Sawamoto et al., 2019[228]). Flavonoids like resveratrol and quercetin can inhibit HDACs (Contreras-Sanzon et al., 2022[65], Kim et al., 2016[151]), leading to the activation of genes involved in neuronal growth and survival, including BDNF. This epigenetic modulation of BDNF expression contributes to the neuroprotective effects of flavonoids. Finally, some flavonoids, such as genistein (found in soy), have been shown to activate estrogen receptors, which are involved in the regulation of BDNF. Estrogen has been shown to increase BDNF expression in the brain (Sohrabji et al., 2006[243]), and polyphenols that mimic estrogen activity may have similar effects (Cipolletti et al., 2018[61]), particularly in the hippocampus, a brain region that is highly responsive to estrogen. Among other mechanisms, some phenolic acids, such as caffeic acid, can activate key signaling pathways involved in neuronal survival and synaptic plasticity. The ERK and phosphoinositide 3-kinase (PI3K) pathways are both involved in the upregulation of BDNF expression (Mohammadi et al., 2018[194]). Studies have shown that caffeic acid can activate these pathways (Ferreira et al., 2019[92]), leading to increased BDNF levels in the brain, particularly in areas involved in learning and memory, like the hippocampus. In addition to their antioxidant effects, phenolic acids can reduce neuroinflammation (Cordeiro et al., 2022[66]), which is often associated with the suppression of BDNF expression. By inhibiting inflammatory cytokines and oxidative stress, phenolic acids may indirectly support the expression of BDNF, enhancing neuronal survival. As observed for phenolic acids, resveratrol is characterized by potent anti-inflammatory properties (Meng et al., 2021[191]). Chronic inflammation is associated with reduced BDNF expression, and resveratrol's ability to reduce neuroinflammation may help restore BDNF levels, particularly in AD-like conditions. Resveratrol is also able to activate SIRT1, a protein deacetylase that plays a key role in cellular stress responses and longevity (Rogina et al., 2024[222]). SIRT1 activation has been linked to increased BDNF expression in the brain, particularly in the hippocampus (Wong et al., 2016[274]). SIRT1 influences BDNF expression by deacetylating key transcription factors, such as CREB, enhancing their ability to bind to DNA and stimulating gene transcription. Resveratrol can also activate AMP-activated protein kinase (AMPK), a central regulator of cellular energy metabolism (DiNicolantonio et al., 2022[79], Ungurianu et al., 2023[261]). AMPK activation has been linked to increased BDNF expression in neurons, especially under conditions of oxidative stress or metabolic dysregulation.

## Role of Polyphenols in Brain Vascular Health

Endothelial cells part of the interior surface of blood vessels play a pivotal role in regulating blood vessel tone, blood flow, and the exchange of molecules between blood and tissues (Trimm et al., 2023[258]). These cells also participate in the immune response by regulating the trafficking of immune cells into tissues (Amersfoort et al., 2022[12]). The vascular and endothelial systems play a crucial role in brain health, particularly in maintaining blood flow, nutrient supply, waste removal, as well as the protection of neurons from harmful substances (Wei et al., 2023[273]). In the brain, endothelial cells are essential constituents of the BBB, which ensure the protection of neurons from toxins, pathogens, and fluctuations in blood composition, also allowing the passage of essential nutrients like glucose and oxygen (Langen et al., 2019[165]). A proper endothelial function in brain vessels is essential for maintaining cerebrovascular integrity, and disruptions in this function can lead to poor blood supply, neuroinflammation, and cognitive decline (Candelario-Jalil et al., 2022[36]). Neurovascular coupling, referring to the ability of blood vessels in the brain to dilate and provide increased blood flow to active neurons, is fundamental for cognitive processes such as learning and memory (Ungvari et al., 2025[262]). In fact, impaired endothelial function can disrupt this process, reducing the ability of brain regions involved in cognition to receive the necessary oxygen and nutrients, especially during periods of high activity (Drew, 2022[80]). Endothelial dysfunction and impaired vascular health are increasingly recognized as contributing factors in neurodegenerative diseases, including AD and vascular dementia (Custodia et al., 2023[70], Fang et al., 2023[86], Gulej et al., 2025[115], Hosoki et al., 2023[126]). Dietary polyphenols have been shown to have significant effects on endothelial function and vascular health (Godos et al., 2019[109]). These effects are especially relevant for preventing cognitive decline, as endothelial dysfunction in brain vessels may lead to reduced cerebral blood flow (CBF), BBB disruption, and neuroinflammation. Polyphenols improve vascular health through a range of molecular mechanisms, and different classes of polyphenols may exert their effects through distinct pathways (Grabska-Kobylecka et al., 2023[112]).

### Vasodilation and cerebral blood flow maintenance

NO is a key vasodilator produced by endothelial cells that plays a crucial role in maintaining healthy blood flow and vascular tone. The endothelial isoform of the enzyme nitric oxide synthase (eNOS) catalyzes the production of NO from the amino acid L-arginine to L-citrulline. NO acts on smooth muscle cells in blood vessel walls to induce vasodilation, increase blood flow, and improve oxygen delivery to brain tissues (Andrabi et al., 2023[15], Tewari et al., 2021[257]). A balance between vasodilators (e.g., NO) and vasoconstrictors (e.g., endothelin-1) release by endothelial cells is necessary for maintaining proper CBF, which is critical for neuronal function, especially in regions involved in cognition, such as hippocampus and prefrontal cortex (Friedman et al., 2022[96], Picon-Pages et al., 2019[207], Wu et al., 2020[276]).

Dietary polyphenols, including flavanols and anthocyanins, can enhance the bioavailability of NO by increasing the activity of eNOS, helping to enhance endothelial-dependent vasodilation (Grosso et al., 2022[114]), thereby improving CBF in areas critical for cognition. Notably, NO is a highly reactive molecule that can be rapidly degraded by reacting with ROS (Takata et al., 2020[254]). Polyphenols, through their antioxidant properties, reduce oxidative stress and decrease the activity of ROS, which in turn helps to preserve the bioavailability of NO (Grabska-Kobylecka et al., 2023[112]). For instance, flavonoids like epicatechin and quercetin scavenge ROS, preventing the degradation of NO and allowing it to exert its vasodilatory effects on brain vessels (Loke et al., 2008[177]). Some polyphenols may also enhance the availability of L-arginine, the substrate for NO synthesis. Through the inhibition of L-arginine degradation promoted by arginase, polyphenols can facilitate increased NO bioavailability, contributing to improved vascular function and blood flow in the brain (Serreli et al., 2023[235]).

### Maintenance of endothelial function

Endothelial cells part of the BBB regulate the transport of molecules into the brain. Any dysfunction in endothelial cells can result in increased permeability of the BBB, which may allow harmful substances to enter the brain and trigger detrimental pathways leading to neuronal damage (Andjelkovic et al., 2023[14], Takata et al., 2021[253]). Oxidative stress represents a key driver of endothelial dysfunction. In the brain context, oxidative stress can impair endothelial cell function, increase BBB permeability, and trigger and/or exacerbate neuroinflammatory phenomena (Kim et al., 2022[154]). Polyphenols have shown the ability to reduce oxidative stress by scavenging free radicals and enhancing the brain's endogenous antioxidant machinery (Feng et al., 2023[91], Kim et al., 2022[154]). Flavonoids (e.g., quercetin, catechins) are characterized by strong antioxidant properties essential for reducing ROS levels and protect endothelial cells from oxidative damage (Li et al., 2023[169]). By modulating the expression of antioxidant enzymes such as SOD, GPx, and catalase, flavonoids help to mitigate oxidative stress and preserve endothelial function (Bernatoniene et al., 2018[25], Deepika et al., 2022[74], Xu et al., 2019[280]). Phenolic acids (e.g., caffeic acid, ferulic acid) exhibit antioxidant effects not only by scavenging free radicals, but also reducing lipid peroxidation in endothelial cells. By protecting endothelial cells from oxidative damage, phenolic acids help preserve vascular function in the brain, including the maintenance of the BBB and neurovascular coupling (Di Giacomo et al., 2022[76]).

Polyphenols exert potent anti-inflammatory effects by modulating key signaling pathways involved in the inflammatory response (Jalouli et al., 2025[137]). Polyphenols such as curcumin, resveratrol, and quercetin have been shown to inhibit the NF-κB pathway, which is a central regulator of inflammation (Mamun et al., 2024[182]). By inhibiting NF-κB activation, polyphenols reduce the expression of pro-inflammatory cytokines (e.g., TNF-α, IL-6) and adhesion molecules (e.g., VCAM-1, ICAM-1), which are involved in the recruitment of immune cells to endothelial cells and the promotion of neuroinflammation (Bhaskar et al., 2016[26], Song et al., 2011[245], Zhang et al., 2020[297]). Polyphenols like resveratrol can also activate Peroxisome Proliferator-Activated Receptors (PPARs), nuclear receptors that regulate inflammation and metabolism. By activating PPARs, polyphenols inhibit the expression of pro-inflammatory genes, thus inflammation of endothelial cells (Enayati et al., 2022[83]). 

## Ability of Polyphenols to Counteract Protein Aggregation

Neurodegenerative diseases, such as AD, are characterized by the aberrant accumulation of misfolded proteins within the brain (Kepp et al., 2023[147]). These proteins can aggregate into larger structures that disrupt cellular function and induce neurodegeneration (Blennow et al., 2018[29]). Two of the most known protein aggregates implicated in AD are amyloid-beta (Aβ) plaques and neurofibrillary tangles, formed by hyperphosphorylated tau protein (Busche et al., 2020[34]). In particular, the formation of amyloid plaques in the brain is toxic for neurons and disrupts normal brain function (Ingelsson et al., 2004[133]). The process of Aβ aggregation involves the formation of very toxic oligomeric species starting from monomeric Aβ peptides (Caruso et al., 2021[42], Caruso et al., 2021[45]), that further aggregate into larger fibrils and plaques (Ahmed et al., 2010[6]). These plaques, surrounded by reactive glial cells such as microglia, are implicated in neuroinflammation, oxidative stress, and synaptic dysfunction, which ultimately contribute to cognitive decline (Heneka et al., 2015[122]).

Dietary polyphenols have been shown to modulate the formation of these toxic aggregates and protect against the progression of neurodegenerative diseases (Hamaguchi et al., 2009[116]). In particular, polyphenols have demonstrated the ability to inhibit the formation of protein aggregates, disrupt pre-formed aggregates, and potentially promote the clearance of misfolded proteins through a multimodal mechanism of action. The neuroprotective effects of polyphenols may vary depending on the class of polyphenol and the specific neurodegenerative disease considered. Many polyphenols can bind directly to the monomeric or oligomeric forms of Aβ, preventing the conformational changes that lead to the formation of toxic aggregates (Hamaguchi et al., 2006[117], Hirohata et al., 2007[123], Ono et al., 2006[199]). Epicatechins and quercetin have been shown to bind directly to Aβ monomers and oligomers, stabilizing them in a non-aggregated state (Jimenez-Aliaga et al., 2011[141], Ono et al., 2003[201]). Other studies have also demonstrated that these flavonoids reduce the formation of amyloid fibrils *in vitro*, as well as the toxic oligomeric species that are believed to be the most neurotoxic forms of Aβ (Alghamdi et al., 2022[9], Bieschke, 2013[27], Hanaki et al., 2016[119], Porat et al., 2006[209], Sternke-Hoffmann et al., 2020[247]). Curcumin, a polyphenol from turmeric, has demonstrated a strong ability to bind to Aβ aggregates, inhibiting the formation of fibrils (Yang et al., 2005[281]). Curcumin also showed to be able to mitigate the toxic effects of Aβ oligomers, for example by inhibiting their ability to assemble and form larger plaques (Ono et al., 2004[200]). Certain polyphenols can also modulate the enzymatic pathways involved in Aβ production. Aβ is derived from the cleavage of the amyloid precursor protein (APP) by two enzymes: β-secretase and γ-secretase (Selkoe, 2001[233]). Polyphenols have been shown to influence the activity of these enzymes, leading to a reduced Aβ production and, in turn, decreased aggregation (Albadrani et al., 2024[8]). Resveratrol has been shown to reduce the activity of β-secretase, the activity of which is essential for the formation of the mature Aβ peptide (Jia et al., 2017[139], Koukoulitsa et al., 2016[157], Skretas et al., 2007[241]). By reducing the amount of Aβ available for aggregation, resveratrol may indirectly prevent the formation of toxic aggregates (Hu et al., 2015[127]). Polyphenols such as EGCG, found in green tea, can prevent the oligomerization of Aβ peptides (Bieschke et al., 2010[28]). EGCG not only binds to Aβ, but also interferes with the conformational changes required for the formation of Aβ oligomers, which are considered to be even more toxic than larger fibrils and plaques (Bieschke et al., 2010[28]). By inhibiting the transition from monomers to toxic oligomers, polyphenols not only reduce the neurotoxicity due to Aβ accumulation, but also preserve the monomeric forms that are believed to exert neuroprotection (Caruso et al., 2019[43]). EGCG also possesses the ability to disrupt the formation of mature Aβ fibrils and plaques, thereby potentially reducing the amyloid burden in the brain (Bieschke et al., 2010[28]). Some polyphenols can also enhance the brain's ability to clear Aβ deposits by stimulating microglial activity. Microglia are the resident immune cells of the brain involved in the clearance of cellular debris, including protein aggregates (Baufeld et al., 2018[23], Heneka et al., 2015[122]). Polyphenols such as epicatechins and resveratrol have been shown to increase microglial phagocytosis of Aβ aggregates, promoting the clearance of these toxic species from the brain. Resveratrol has also been shown to modulate the p62/SQSTM1 pathway, which is involved in autophagic clearance of protein aggregates, including Aβ (Kim et al., 2013[152], Li et al., 2011[170], Marambaud et al., 2005[184], Renaud et al., 2015[220]). By enhancing autophagic degradation of Aβ, resveratrol helps to reduce the accumulation of toxic amyloid plaques in the brain (Ghobeh et al., 2014[103]).

As previously mentioned, in addition to Aβ plaques, neurofibrillary tangles formed by hyperphosphorylated tau represent another key pathological feature of AD and other tauopathies, such as frontotemporal dementia and progressive supranuclear palsy (Rademakers et al., 2004[211]). Tau is a microtubule-associated protein that stabilizes microtubules within neurons (Goedert et al., 2005[110], Reddy, 2011[216]). Under pathological conditions, tau becomes hyperphosphorylated and forms insoluble aggregates that disrupt microtubule function, impair cellular transport, contributing to neurodegenerative phenomena (Kanaan et al., 2013[144]). Polyphenols can interfere with tau aggregation, preventing the toxic effects of tangles in several ways (Zheng et al., 2019[302]). One of the key events in tau aggregation is its hyperphosphorylation, which alters the protein's structure and promotes its aggregation (Wegmann et al., 2021[272]). Certain polyphenols have been shown to inhibit the kinases responsible for tau phosphorylation (Zheng et al., 2019[302]). For example, curcumin has been demonstrated to reduce tau phosphorylation, thus the formation of toxic tau aggregates, by inhibiting the activity of glycogen synthase kinase-3β (GSK-3β) (Wang et al., 2019[267]). Resveratrol has also been shown to reduce tau hyperphosphorylation by modulating AMPK signaling, which in turn regulates tau kinase activity (Porquet et al., 2013[210]). Polyphenols such as EGCG have been shown to directly interact with tau, inhibiting the formation of tau aggregates. EGCG can bind to tau monomers and prevent their aggregation into oligomers and fibrils (Sonawane et al., 2020[244]). Studies have suggested that EGCG can disrupt tau fibrils and/or prevent the formation of neurotoxic tau aggregates. Epicatechins can also inhibit tau aggregation by stabilizing tau in its monomeric or non-toxic oligomeric form. As observed with Aβ, polyphenols have been shown to enhance the clearance of tau aggregates (Chesser et al., 2016[59]). The activation of the autophagy-lysosomal pathway as well as the involvement of microglial cells in tau clearance are important mechanisms through which polyphenols may reduce tau load in the brain (Marambaud et al., 2005[184]). Resveratrol has been shown to activate sirtuins, which are involved in regulating protein degradation pathways, including autophagy (Hubbard et al., 2014[132]). This activation of autophagy helps to promote the clearance of tau aggregates and reduce tau-related neurodegeneration (Chesser et al., 2016[59], Schweiger et al., 2017[232]). Finally, neuroinflammation plays a significant role in the propagation of tau pathology (Chen et al., 2023[53]). Polyphenols such as quercetin and curcumin reduce neuroinflammation by inhibiting pro-inflammatory signaling pathways like NF-κB and JNK, which have been associated with the tau aggregation (Simunkova et al., 2019[239], Suganthy et al., 2016[249], Zaplatic et al., 2019[291]). Therefore, the reducing of neuroinflammation by polyphenols may slow down the progression of tau-related diseases (Zheng et al., 2019[302]).

## Epigenetic Interactions of Polyphenols in the Brain

**Figure 5:**
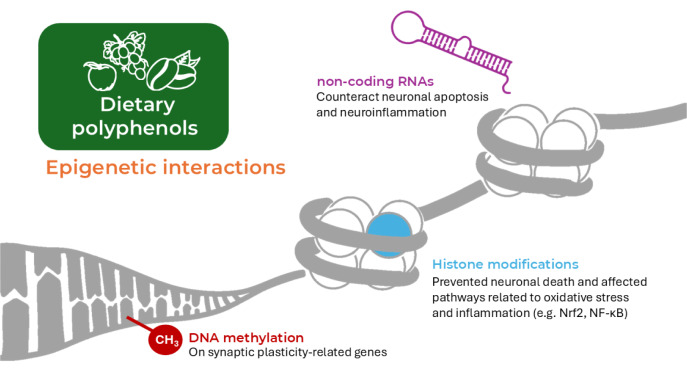
Dietary polyphenols have epigenetic interactions in the brain. In particular, polyphenols can contribute to histone modifications (leading to the prevention of neuronal death and affecting pathways related to oxidative stress and neuroinflammation such as NF-κB and Nrf2), DNA methylation (affecting pathways related to synaptic plasticity), and non-coding RNAs (counteracting neuronal apoptosis and neuroinflammation).

Besides all the proposed effects in the brain context, polyphenols are also able to influence cellular functions through epigenetic regulation. Several studies have demonstrated that dietary polyphenols can modulate DNA methylation, histone modifications, and non-coding RNAs (Figure 5(Fig. 5)), thereby contributing to their long-term protective effects (Borsoi et al., 2023[31], Fiore et al., 2025[93]). 

In a murine model, a bioavailable polyphenol preparation (BDPP) containing resveratrol was shown to alter DNA methylation patterns, including in introns, UTRs, and exons, of hippocampal genes. These changes were linked to transcriptional shifts in synaptic plasticity-related genes, mediated by differential expression of DNA methyltransferases (DNMTs) and ten-eleven translocation methylcytosine dioxygenases (TETs) enzymes (Frolinger et al., 2018[97]). In an *in vitro* model of brain ischemia, a polyphenol-enriched micronutrient mixture (including EGCG, resveratrol, and quercetin) prevented neuronal death in primary mouse cortical neurons exposed to oxygen-glucose deprivation (Faggi et al., 2019[84]). The treatment with the mixture rich in polyphenols exerted epigenetic regulation by modulating the acetylation at Lys310 of NF-κB/RelA (p65 subunit of the NF-κB transcription factor), and the histone H3 acetylation at the Bim promoter, a pro-apoptotic target of ac-RelA in brain ischemia. Polyphenols such as curcumin have also been reported to regulate histone acetyltransferases (HATs) and histone deacetylases (HDACs), leading to transcriptional reprogramming in pathways related to oxidative stress and inflammation, including Nrf2 and NF-kB (Hassan et al., 2019[121]). Moreover, polyphenols affect non-coding RNAs: in AD models, polyphenols have been demonstrated to counteract neuronal apoptosis and neuroinflammation by modulating specific microRNAs, thereby influencing Aβ metabolism and inflammatory signaling pathways (Zhou et al., 2025[303]). The additional ability to interact with the epigenome, beyond the intrinsic antioxidant and anti-inflammatory properties of polyphenols, may represent a significant advantage in enhancing their neuroprotective potential.

## Main Limitations of Current Evidence

While animal studies have provided valuable insights into the therapeutic potential of polyphenols in the brain, there are several possible limitations worth of mention. First of all, the bioavailability of polyphenols in animals is often much higher than in humans due to differences regarding metabolism, absorption, and processing (Scalbert et al., 2000[229]). As a result, the amount of polyphenols used in animal studies may be much higher than those achievable through human dietary intake. Despite that, even in animals, the amount of polyphenols that reach the brain after oral consumption is often quite low due to the poor solubility coupled to the restrictive nature of the BBB. While some polyphenols can cross the BBB, the quantities that reach the brain are typically insufficient to produce significant effects without using very high and possibly toxic doses or specialized drug delivery systems (Grabska-Kobylecka et al., 2023[112]). Additionally, humans and animals metabolize polyphenols differently, which can lead to variations in the bioavailability and activity of polyphenols as well as in the formation of their metabolites (van Duynhoven et al., 2011[263]), which could influence the effectiveness of polyphenols in the brain.

## Conclusions

In conclusion, recent preclinical and clinical evidence suggests that polyphenols may exert neuroprotective and pro-cognitive effects by modulating different cellular pathways involved in oxidative stress and neuroinflammation. Despite these promising findings, limitations regarding the bioavailability of polyphenols in humans and the ability to cross the BBB along with the potential differences in metabolism and metabolites formation remain significant challenges. Future translational studies should be devoted to overcome these limitations allowing to fully unveil the therapeutic potential of polyphenols in the treatment of cognitive disorders.

## Declaration

### Acknowledgments

F.G. and G.G. were supported by the project entitled “ON Foods-Research and innovation network on food and nutrition Sustainability, Safety and Security-Working ON Foods” funded under the National Recovery and Resilience Plan (NRRP), Mission 4 Component 2 Investment 1.3-Call for tender No. 341 of 15 March 2022 of Italian Ministry of University and Research funded by the European Union-NextGenerationEU; Project code PE00000003, Concession Decree No. 1550 of 11 October 2022 adopted by the Italian Ministry of University and Research.

### Conflict of interest

The authors declare no conflict of interest.

### Artificial Intelligence (AI) - Assisted Technology

The authors declare that they have not used any type of generative artificial intelligence for the writing of this manuscript, nor for the creation of images, graphics, tables, or their corresponding captions.

## Figures and Tables

**Figure 1:**
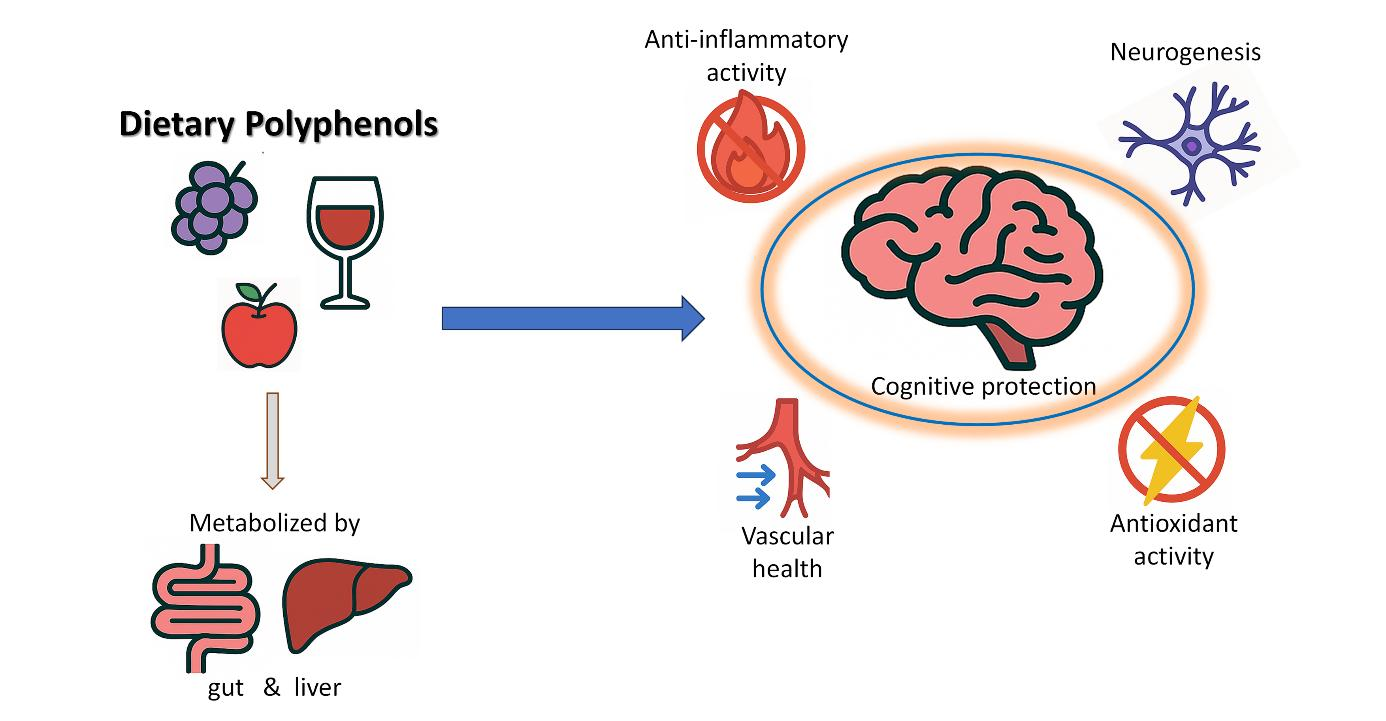
Graphical abstract
